# Alien introgression and morpho-agronomic characterization of diploid progenies of *Solanum lycopersicoides* monosomic alien addition lines (MAALs) toward pre-breeding applications in tomato (*S. lycopersicum*)

**DOI:** 10.1007/s00122-020-03758-y

**Published:** 2021-01-02

**Authors:** Puneet Kaur Mangat, Junghyun Shim, Ritchel B. Gannaban, Joshua J. Singleton, Rosalyn B. Angeles-Shim

**Affiliations:** Department of Plant and Soil Science, College of Agricultural Sciences and Natural Resources, Texas Tech University, Lubbock, TX 79409-2122 USA

## Abstract

**Key message:**

Alien introgressions that were captured in the genome of diploid plants segregating from progenies of monosomic alien addition lines of *S. lycopersicoides* confer novel phenotypes with commercial and agronomic value in tomato breeding.

**Abstract:**

*Solanum lycopersicoides* is a wild relative of tomato with a natural adaptation to a wide array of biotic and abiotic challenges. In this study, we identified and characterized diploid plants segregating from the progenies of monosomic alien addition lines (MAALs) of *S. lycopersicoides* to establish their potential as donors in breeding for target trait improvement in tomato. Molecular genotyping identified 28 of 38 MAAL progenies having the complete chromosome complement of the cultivated tomato parent and limited chromosome introgressions from the wild *S. lycopersicoides* parent. Analysis of SSR and indel marker profiles identified 34 unique alien introgressions in the 28 MAAL-derived introgression lines (MDILs) in the genetic background of tomato. Conserved patterns of alien introgressions were detected among sibs of MDILs 2, 3, 4 and 8. Across MDILs, a degree of preferential transmission of specific chromosome segments was also observed. Morphologically, the MDILs closely resembled the cultivated tomato more than *S. lycopersicoides*. The appearance of novel phenotypes in the MDILs that are lacking in the cultivated parent or the source MAALs indicates the capture of novel genetic variation by the diploid introgression lines that can add commercial and agronomic value to tomato. In particular, screening of representative MDILs for drought tolerance at the vegetative stage identified MDIL 2 and MDIL 11III as drought tolerant based on visual scoring. A regulated increase in stomatal conductance of MDIL 2 under drought stress indicates better water use efficiency that allowed it to survive for 7 days under 0% moisture level.

**Supplementary Information:**

The online version contains supplementary material available at 10.1007/s00122-020-03758-y.

## Introduction

Tomato (*Solanum lycopersicum* L.) is one of the most important vegetable crops, with global production reaching up to 182.3 million tons in 2019 (FAOSTAT 2019). Like most cultivated plants, tomato has undergone rigid selection for only a small number of key agronomic traits that include fruit yield and quality during domestication. The stringent criteria for artificial selection, coupled with a strictly autogamous means of reproduction, have created a bottleneck that severely narrowed the genetic base of the crop. With less than 5% of genetic variation found in its wild relatives (Miller and Tanksley 1990; Bai and Lindhout 2007), tomato stands vulnerable to the emerging agricultural challenges brought about by the compounded effects of intensive farming and rapidly changing climate. Ensuring the sustainability of tomato production amidst a worsening agro-environment will require the expansion of the genetic base of the crop through the re-introduction of genetic variation from diverse germplasm that can provide novel and durable forms of adaptation against a multitude of biotic and abiotic stresses.

Wild relatives of crops are a rich reservoir of naturally occurring genetic variation for tolerance to environmental stresses and resistance to pests and pathogens. In tomato, the nearly 80 years of genetic improvement has greatly relied and benefited from the introgression of natural alleles regulating desirable traits from its wild relatives (Rick and Chetelat 1995; Bai 2017; Gur and Zamir 2004).

*S. lycopersicoides* from the section *Lycopersicoides* of the genus *Solanum* is a wild nightshade species that is distantly related to tomato. It is native to the western slopes of the Andes cordillera bordering Chile and Peru and thrives at altitudes of up to 3800 m above sea level (Rick 1976). Aside from tolerance to cold, drought and salinity (Wolfe et al. 1986; Li et al. 2011; Zhang et al. 2013), *S. lycopersicoides* has also known resistance to Dipteran and Lepidopteran pests (Rick 1976; Canady et al. 2005) and to a wide array of pathogenic fungi (e.g., *Botrytis cinerea* and *Phytophthora parasitica*), bacteria (e.g., *Xanthomonas campestris* and *Clavibacter michiganensis* subsp. *michiganensis*) and viruses (e.g., Tomato yellow leaf curl virus, Tomato mosaic virus, Tomato crinivirus and Cucumber mosaic virus) that commonly infect tomato (Chetelat et al. 1997; Davis et al. 2009; Perez et al. 2011; Phills et al. 1977a, b; Zong et al. 2012).

Sizable efforts to harness the rich genetic variation conferring wide adaptability to *S. lycopersicoides* have led to the successful development of pre-breeding stocks of the wild nightshade in the form of early backcross populations, chromosome segment substitution lines (CSSLs) and monosomic alien addition lines (MAALs) (Gradziel et al. 1989; Chetelat et al. 1997,1998; Canady et al. 2005). CSSLs are a set of introgression lines, usually in the genetic background of an elite cultivar, that represent the whole genome of a donor line (i.e., wild species) in small and contiguous chromosome segments (Besho-Uehara et al. 2017; Furuta et al. 2016; Shim et al. 2010). MAALs, on the other hand, are pre-breeding lines that carry an additional chromosome from the wild donor alongside the full chromosome complement of the cultivated, recipient parent (2*n* + 1) (Khush 2010). This distinctive feature of MAALs makes them highly suitable for assigning alien traits to specific chromosomes of the wild donor, dissecting wild genomes into individual chromosome entities in a functional genomic background, and identifying novel genes underlying traits of agronomic importance (Khush 2010; Wang et al. 2016; Narain et al. 2016). For introgressive breeding applications, however, the utilization of MAALs is largely restricted by the unstable transmission of the additional chromosome, as well as the potential loss of chromosome integrity due to homeologous recombinations (Miller and Tanksley 1990; Chetelat et al. 1998).

Progenies of self-pollinated MAALs have been reported to segregate at varying frequencies into plants with the additional chromosome and those having the normal chromosome complement of the cultivated parent. In rice for example, *Oryza latifolia* MAAL progenies have been shown to segregate into both MAALs and fertile diploid lines at a ratio of 1:3, with the diploids mostly resembling the overall morphology of the cultivated parent. Genotyping of the identified diploids mapped distinct patterns of alien introgressions in the genetic background of the recurrent parent which were later associated with novel agronomic traits lacking in cultivated rice (Angeles-Shim et al. 2014, 2020).

Similarly, analysis of the stability of chromosome transmission in progenies of *S. lycopersicoides* MAALs established the presence of diploid plants at a considerably high frequency of 75–100%. In fact, MAALs 1 and 6 which transmitted the additional alien chromosome at very low rates of 0–2% produced only diploid progenies (Chetelat et al. 1998). As in the case of rice, diploid lines segregating from the *S. lycopersicoides* MAALs will have maintained wild chromosome segment introgressions for as long as they did not compromise the fitness of the plants. These diploid introgression lines constitute an important but overlooked genetic resources that can potentially contribute in reinvigorating the depleted genetic base of tomato. To facilitate the utilization of these exotic germplasm in breeding for value-added traits in tomato, systematic genotyping and phenotyping that will map the alien introgressions in the genome of the diploid progenies and evaluate the effects of such introgressions on the morphological and agronomic fitness of the recurrent genetic background are necessary.

In this study, we identified alien introgressions in the diploid progenies of *S. lycopersicoides* MAALs and estimated their location in the genome using DNA markers that are specific to both tomato and the wild nightshade species. Morphological and agronomic characterization showed novel phenotypes in the diploid lines that are due to the alien introgressions. Associations between the observed phenotypes and unique introgressions in specific diploid lines identified putative loci controlling drought tolerance in *S. lycopersicoides*. The newly characterized germplasm reported in this study provides basis for future research on the identification, mapping, and transfer to elite tomato backgrounds of genes/QTLs underlying traits of agronomic interest from the wild nightshade species.

## Materials and methods

### Plant materials

Seeds of tomato (cv. Vendor Acc. LA3122), *S. lycopersicoides* (Acc. LA1964) and MAALs 2, 3, 4, 5, 7, 8, 9, 10, 11 and 12 of the wild nightshade species were provided by the Tomato Genetics Resource Center (TGRC) of the University of California, Davis (http://tgrc.ucdavis.edu). LA3122 was the tomato accession used to generate the MAALs (Chetelat et al. 1998). MAALs 1 and 6 were excluded from the study due to the very low transmission of the additional chromosome in these MAALs.

Eight seeds per MAAL were directly sown and germinated in one-liter plastic pots containing conventional growth media composed of 45–50% composted pine bark, vermiculite, Canadian sphagnum, peat moss, perlite and dolomitic limestone. Four weeks after emergence, leaf tissues were sampled from each plant and used to extract genomic DNA following a modified CTAB method (Murray and Thompson 1980). All plant materials were maintained up to maturity in a greenhouse at the horticultural gardens of the department of plant and soil science at Texas Tech University.

### Genotyping of *S. lycopersicoides* MAAL progenies and identification of diploid segregants

A total of 445 DNA markers that include 100 simple sequence repeats (SSR) that are specific to tomato, as well as 345 SSR and insertion/deletion (indel) markers that are specific to *S. lycopersicoides* were screened for their ability to amplify polymorphic targets in the tomato and *S. lycopersicoides* genomes. The tomato SSRs were selected from a marker list available from the Kazusa Tomato Marker Database (http://marker.kazusa.or.jp/Tomato/) (Table S1). The *S. lycopersicoides-*specific DNA markers were designed following specifications for standard primer design using an in-house analysis of a draft assembly of the *S. lycopersicoides* (Acc. LA1964) genome (Mangat et al. 2020). Synthesis of all primer pairs was outsourced to Sigma-Aldrich, USA. Markers that were able to differentiate between the genomes of tomato and *S. lycopersicoides* were used to detect alien chromosome introgressions in the MAAL progenies following a standard protocol for PCR (Shim et al. 2015). All amplicons were resolved in 3% agarose gel in 1X Tris–Borate-EDTA (TBE) buffer. Assignment of alien introgressions was based on comparisons with the molecular weight of target amplicons in tomato and *S. lycopersicoides*. Alien introgressions were mapped in the chromosomes based on the physical location of the markers flanking the amplified *S. lycopersicoides-*specific fragments. Progenies showing heterozygous marker alleles (i.e., one from the *S. lycopersicoides* and another from the tomato parent) were considered MAALs if the *S. lycopersicoides* fragment was amplified from the same chromosome as the extra chromosome carried by the MAAL from which the progenies were derived. Otherwise, the lines were designated as MAAL-derived introgression lines or MDILs. The MDILs are diploid segregants of the MAALs having *S. lycopersicoides* chromosome introgressions in the background of the cultivated tomato.

To confirm the stability of *S. lycopersicoides* chromosome transmission, five seeds from each of the identified MDILs were directly sown and germinated in one-liter pots containing conventional potting mix and maintained up to maturity in the greenhouse. Following the procedure described previously for DNA extraction, PCR and gel electrophoresis, the MDIL progenies were genotyped using the same SSRs and indels that identified the *S. lycopersicoides* introgressions in the original MDILs.

### Morpho-agronomic characterization of the *S. lycopersicoides* MDILs

The morphological characteristics of the identified MDILs and their progenies were catalogued based on five plants each using the Standard Descriptors for Tomato (IPGRI 1996). Data were recorded for plant growth type (i.e., determinate, semi-determinate or indeterminate), leaf type (i.e., standard, dwarf, potato-leaf, peruvianum, pimpinellifolium or hirsutum), leaf attitude (i.e., semi-erect, horizontal or drooping) and style position (inserted, same level as stamen, slightly exserted or highly exserted). Leaf traits were catalogued using the fifth leaf sampled from each plant at the onset of flowering. Fruit shape, size, size homogeneity, weight (g) and locule number were determined based on five fruits per plant that were harvested at full maturity. Fruit size was based on cross-sectional diameter and was classified as very small (< 3 cm), small (3–5 cm), intermediate (5.1–8 cm), large (8.1–10 cm) or very large (> 10 cm). Fruits of MDILs were cut cross-wise and scanned using an Epson Perfection V600 photo scanner. The scanned images were then used to determine fruit size and number of locules per fruit.

Screening for drought tolerance was carried out using five plants for each representative MDIL that segregated from each of the 10 MAALs, along with the cultivated tomato Acc. LA3122. Seeds were sown and germinated in half-liter pots containing conventional growth media supplemented with NPK fertilizers and maintained under ambient temperature in the laboratory. For four weeks following emergence, seedlings were watered everyday up to full saturation capacity of the growth media. Water was withheld thereafter and moisture content in the substrate was monitored daily using the FieldScout TDR300 (Spectrum Technologies Inc., USA) until it reached approximately 0%. The responses of the plants to gradual water loss were evaluated based on visual scoring of the plants at 0% moisture content. Plants that remained turgid at 0% moisture content were scored as tolerant and those that wilted were scored susceptible.

To validate the responses of the MDILs that showed drought tolerance based on visual scoring, similar drought experiments in one-liter pots were set up in three technical replicates under laboratory conditions. Aside from visual scoring, physiological evaluation of the responses of the select MDILs to the stress was also carried out. Stomatal conductance and chlorophyll content were measured from 5 leaves per plant using the SC-1 leaf porometer (Decagon Devices, WA, USA) and MC-100 chlorophyll content meter (Apogee, UT, USA), respectively, 12 days after the last watering of the plants when moisture content in the growth media was approximately 25%.

## Results

### MAALs segregate into MAALs and MDILs with distinct patterns of alien introgressions

Of the 445 DNA markers used to genotype the MAAL progenies, 328 were able to amplify targets in both tomato and *S. lycopersicoides* genomes. Of these, 154 were polymorphic including four SSRs (TGS 1790, TGS 1838, TGS 12,842 and TGS 1264) that are based on the tomato genome, and 52 SSRs and 98 indels that were designed based on the *S. lycopersicoides* genome (Table S2). The DNA markers are distributed across the 12 tomato chromosomes at an average interval of 5 Mb.

A total of 38 plants segregating from the 10 *S. lycopersicoides* MAALs were genotyped using the polymorphic SSR and indel markers. The number of segregants that were genotyped per MAAL ranged from 2 to 6, depending on the survivability of the progenies in the greenhouse (Table 1). DNA marker profiling showed the progenies of MAALs 9, 10, 11 and 12 segregating into both MAALs and MDILs, with the MDILs occurring at a frequency of 25–66%. Aside from the additional chromosome, each MAAL carries small alien introgressions in its diploid chromosome complement. Conversely, MAALs 2, 3, 4, 5, 7 and 8 produced only MDILs. A single progeny segregating from MAAL 7 showed no identifiable *S. lycopersicoides* introgressions in any of its twelve chromosomes. Overall, 28 MDILs were identified out of the 38 plants derived from the MAALs.

**Table 1:** Classification of progenies segregating from *S. lycopersicoides* MAALs based on alien introgression patterns

MAAL source	Plant no	Chromosome introgression from *S. lycopersicoides*	Segregant type
MAAL 2	1	1, 2, 4, 5, 7	MDIL 2
	2	1, 2, 4, 5, 7	MDIL 2
	3	1, 2, 4, 5, 7	MDIL 2
	4	1, 2, 4, 5, 7	MDIL 2
MAAL 3	1	5	MDIL 3
	2	5	MDIL 3
MAAL 4	1	5	MDIL 4I
	2	5	MDIL 4I
	3	5	MDIL 4I
	4	5	MDIL 4I
	5	5, 6	MDIL 4II
MAAL 5	1	5, 6, 9	MDIL 5I
	2	5, 9	MDIL 5II
	3	5, 6, 8, 9, 12	MDIL 5III
MAAL 7	1	-	-
	2	5	MDIL 7
MAAL 8	1	4, 5	MDIL 8I
	2	4, 5	MDIL 8I
	3	4, 5	MDIL 8I
	4	1, 4, 5	MDIL 8II
	5	4, 5	MDIL 8III
MAAL 9	1	5	MDIL 9
	2	5 + chr 9	MAAL 9I
	3	1, 2, 5 + chr 9	MAAL 9II
MAAL 10	1	5, 9	MDIL 10
	2	5 + chr 10	MAAL 10I
	3	5 + chr 10	MAAL 10II
	4	1, 5, 9, 12 + chr 10	MAAL 10III
MAAL 11	1	5, 6	MDIL 11I
	2	5, 6	MDIL 11II
	3	5, 6	MDIL 11III
	4	1, 5, 6	MDIL 11IV
	5	4, 5, 6 + chr 11	MAAL 11I
	6	5, 6, 8 + chr 11	MAAL 11II
MAAL 12	1	4, 5, 6	MDIL 12I
	2	5, 6	MDIL 12II
	3	4, 5, 6, + chr 12	MAAL 12I
	4	5, 12 + chr 12	MAAL 12II

**Fig. 1:**
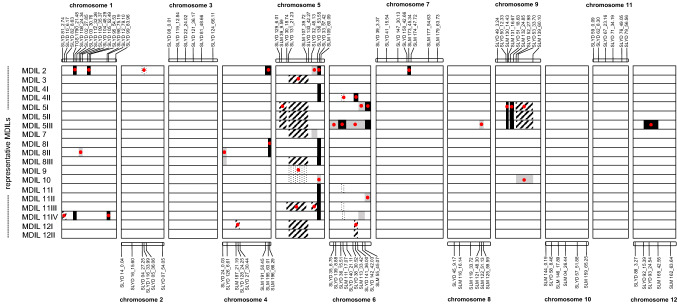
Graphical genotype of MDILs representing 19 distinct combinations of chromosome segment introgressions from *S. lycopersicoides*. Black and gray bars represent homozygous introgressions from *S. lycopersicoides* and heterozygous introgression from both parents, respectively. Striped and dotted bars represent homozygous and heterozygous, non-parental introgressions, respectively. Red dots indicate the 34 unique wild introgressions detected across all the identified MDILs that segregated from the MAALs. Representative, polymorphic markers used to genotype the MDILs are illustrated. Numbers following the marker name indicate the physical position (Mb) of the markers in the chromosome

**Fig. 2:**
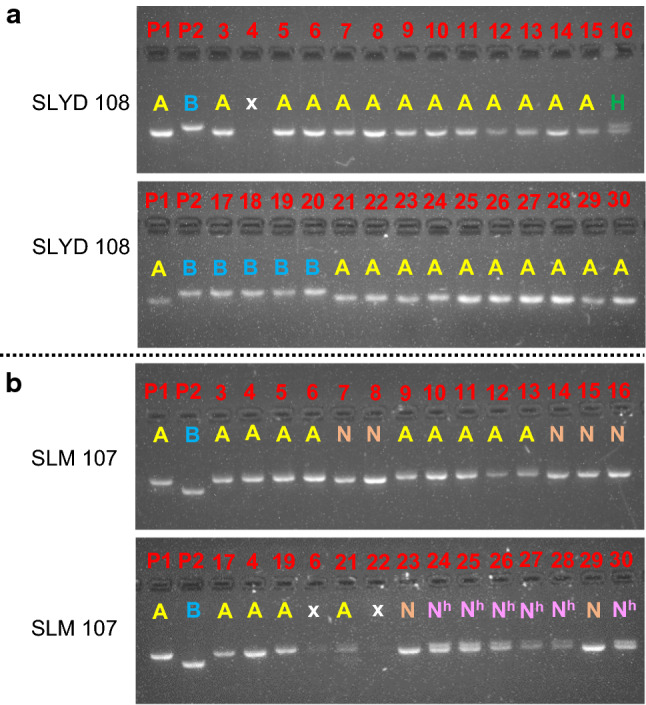
Banding patterns of amplified markers in the MDILs. Banding patterns of the (**a**) indel marker SLYD 108 and (**b**) SSR marker SLM 107 in tomato (1), *S. lycopersicoides* (2) and the MDILs (3–30). (**c**) The letters A and B indicate homozygous alleles, whereas the letter H indicates heterozygous alleles from P1 and P2, respectively. *N* signify homozygous, non-parental bands, whereas *N*^*h*^ indicates heterozygous allele from P1 and a non-parental band, *N*. Nos. 3–6 = representatives of MDIL 2, Nos. 7–8 = representatives of MDIL 3, Nos. 9–12 representatives of MDIL 4I, No. 13 = representative of MDIL 4 II, No. 14 = representative of MDIL 5I, No. 15 = representative of MDIL 5II, No. 16 = representative of MDIL 5III, Nos. 17–18 = representatives of MDIL 7, Nos. 19, 21–22 = representatives of MDIL 8I, Nos. 20, 23, 24, 25, 26, 27, 28, 29 and 30 = representative of MDIL 8II, MDIL 8III, MDIL 9, MAAL 9I, MAAL 9II, MDIL 10, MAAL 10I, MAAL 10II and MAAL 10III, respectively. x = non-amplification or unclear band

Analysis of the genotype data of the 28 MDILs detected a total of 34 unique alien introgressions that ranged from 4.34 Mb in chromosome 9 to 32.12 Mb in chromosome 9 (Table S3). The chromosome segment introgressions were in the form of homozygous *S. lycopersicoides* alleles, heterozygous alleles coming from both parents and/or homozygous or heterozygous, non-parental alleles (Fig. 1, Fig. 2). Most MDILs carry a unique set of chromosome segments from *S. lycopersicoides* despite coming from the same MAAL although in some cases, similar patterns of alien introgressions were observed across sibs as was noted in progenies produced by selfing MAALs 2, 3, 4 and 8 (Table 1). Of the 28 MDILs, 19 exhibited distinct patterns of *S. lycopersicoides* introgression (Fig. 1). MDIL 5III had the most number of introgressions in chromosomes 5, 6, 8, 9 and 12, whereas MDILs 3, 4I and 9 showed just a single introgression each in chromosome 5. All MDILs have an introgression in chromosome 5, whereas none were detected in chromosomes 3, 10 and 11.

Self-pollination of the MDILs in the greenhouse produced seeded fruits except for a few, non-loculed berries generated by MDIL 12 (Fig. 4c). Genotyping of the progenies of the original MDILs confirmed the stability of *S. lycopersicoides* chromosome transmission, including the heterozygous alleles in chromosomes 1, 4, 5, 6, 8, and 9, and the non-parental bands in chromosomes 2, 5 and 6.

### *S. lycopersicoides* chromosome segment introgressions created novel phenotypes in the MDILs

**Fig. 3:**
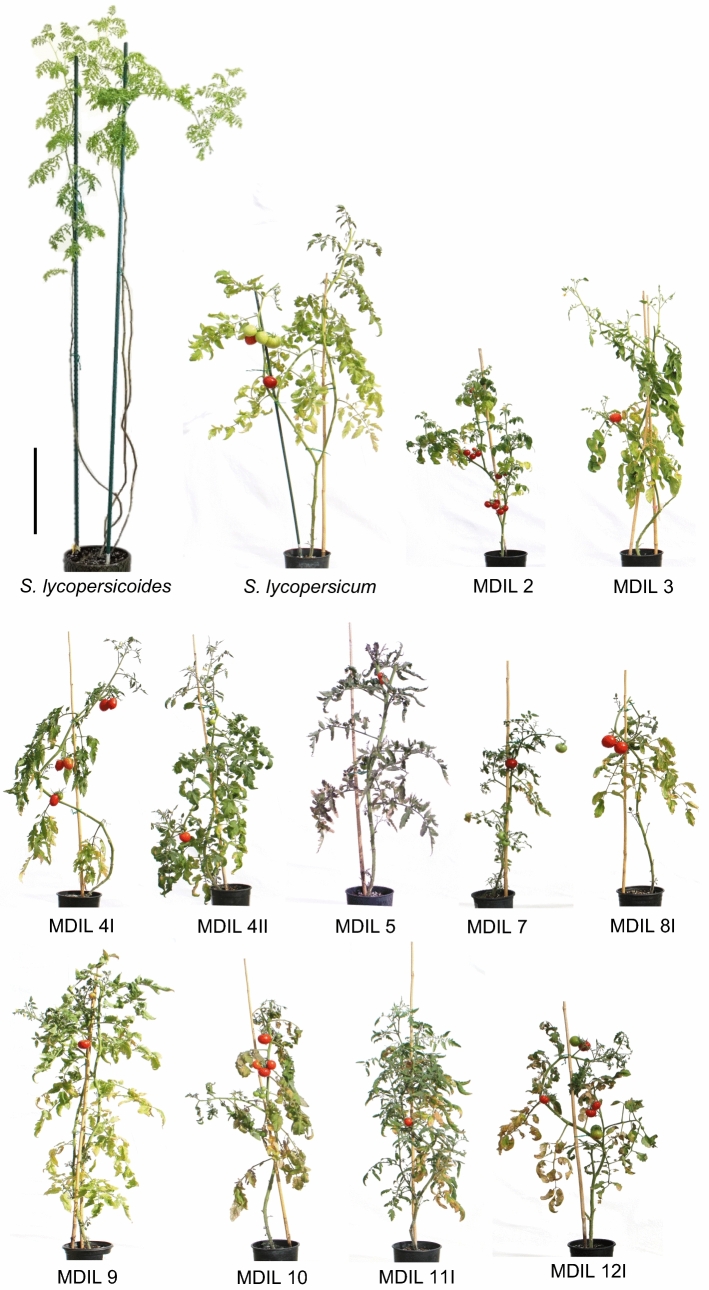
Gross morphology of representative MDILs that segregated from each MAAL. Bar = 30 cm

Differences in the gross morphology of representative MDILs were observed, with the lines closely resembling the overall habit of the cultivated tomato parent and not that of the *S. lycopersicoides* parent (Fig. 3). All MDILs were indeterminate and continued to set fruits under greenhouse conditions. Except for MDIL 2 which remained compact and bushy, all other MDILs continued growing in height and required staking to prevent the plants from falling over (Table 2, Fig. 3).

**Table 2:** Morphometric characteristics of the MDILs scored using the standard descriptors for tomato (IPGRI, 1996)

Line name	Growth type	Leaf type	Leaf attitude	Style position	Fruit descriptors				
					Shape	Size	Weight^a^ (g)	Size homogeneity	Locule number
Acc LA 3122	Indeterminate	Standard	Semi-erect	Inserted	Round	Intermediate	43.10	High	3
MDIL 2	Indeterminate	Potato-like	Semi-erect	Inserted	Round	Very small	9.47	High	2–4
MDIL 3	Indeterminate	Potato-like	Drooping	Inserted	Slightly flattened	Small	39.66	High	4
MDIL 4I	Indeterminate	Peruvianum	Drooping	Inserted	Cylindrical	Small	33.95	Intermediate	2–3
MDIL 5I	Indeterminate	Hirsutum	Horizontal	Inserted	Slightly flattened	Small	22.50	High	3–4
MDIL 7	Indeterminate	Standard	Semi-erect	Inserted	Round	Intermediate	36.49	Intermediate	2–3, 6
MDIL 8I	Indeterminate	Standard	Semi-erect	Exserted	Heart-shaped	Small	32.74	High	2–3
MDIL 9	Indeterminate	Standard	Semi-erect	Inserted	Oblate	Small	35.26	High	3
MDIL 10	Indeterminate	Standard	Semi-erect	Inserted	Round	Small	24.15	High	6
MDIL 11I	Indeterminate	Hirsutum	Semi-erect	Inserted	Round	Small	30.74	High	2, 6
MDIL 11II	Indeterminate	Standard	Semi-erect	Inserted	Cylindrical	Small	–	High	2, 6
MDIL12I	Indeterminate	Standard	Semi-erect	Exserted	Round	Small	24.37	High	0, 3–4

^a^Shows average weight for a single fruit

**Fig. 4:**
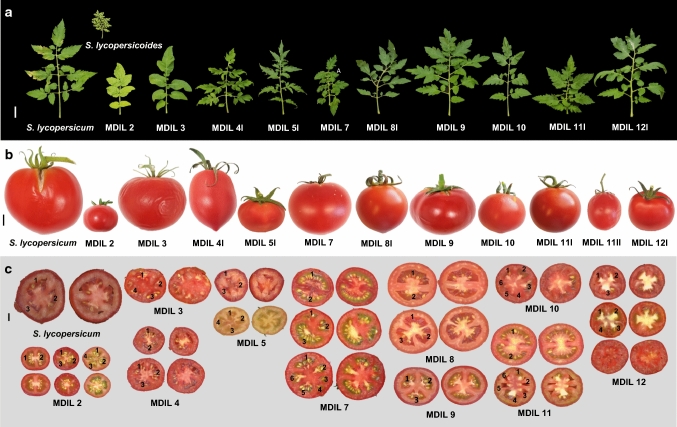
Novel phenotypes created by *S. lycopersicoides* introgressions in the MDILs. (**a**) Variations in the color, shape, size and dissection of leaves of tomato Acc. LA3122, *S. lycopersicoides* and the MDILs. Bar = 5 cm (**b**) Variations in the shape and size of fruits of tomato Acc. LA3122 and the MDILs. Bar = 1 cm. (**c**) Variations in the locule number of fruits of tomato Acc. LA3122 and the MDILs. Bar = 1 cm

Most MDILs have the same standard, semi-erect leaves as the tomato parent with slight variations in the overall size and degree of dissection. MDILs 2 and 3 have potato-like leaves, although those of MDIL 2 were distinctly yellowish-green and semi-erect, whereas those of MDIL 3 were dark green and drooping. Leaves of MDIL 5I and 11I were of the hirsutum type, whereas those of MDIL 4I were of the peruvianum type (Table 2, Figs. 3,  4a). A distinct anthocyanin coloration on the abaxial surface of the leaves was observed in MDIL 5, giving the whole plant an overall purple tinge (Fig. 3). Except for MDIL 8I and 12I which have exserted styles, all lines exhibited an inserted style (Table 2).

Wide variability was also observed in the fruits of the MDILs in terms of shape, size and weight, although homogeneity in fruit size within a plant was generally high. Fruit weight generally corresponded with size which ranged from small to intermediate. MDIL 2 produced fruits which are distinctly small, round and bright red in color that are similar to cherry tomatoes. Locule number differed among fruits harvested within the same plant and ranged from 0 to 6 across the MDILs (Table 2, Fig. 4c). Deviations from the round fruit shape of the cultivated tomato were observed in fruits produced by MDILs 3, 4, 5I, 8I, 9 and 11II (Table 2, Fig. 4b).

At a constant temperature of 25 °C, *S. lycopersicoides* produced buds that failed to develop into full flowers even after more than two months, hence the absence of fruit specimen for comparison with those of the MDILs.

### MDIL 2 and MDIL 11III acquired tolerance to gradual water deficit at the early vegetative stage from *S. lycopersicoides* chromosome segment introgression

**Fig. 5:**
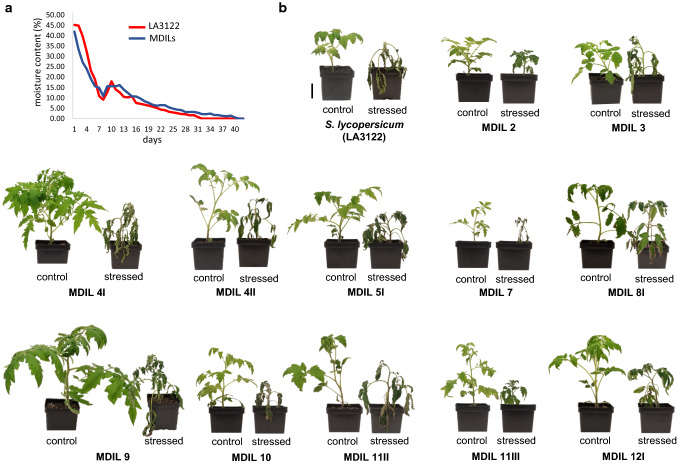
Visual responses of representative MDILs to gradual water deficit under laboratory conditions. (**a**) Average moisture content of potting mix planted with the tomato Acc. LA3122 and MDILs. Day 1 represent the last day the plants were watered to full saturation capacity. (**b**) Gross morphology of tomato and MDILs 7 days after the moisture content of the potting mix reached 0%. MDIL 2 and MDIL 11III showed no signs of wilting or curling of leaves. Bar = 10 cm

Initial drought screening under laboratory conditions of representative MDILs that segregated from each MAAL identified MDIL 2 and MDIL 11III to have tolerance to gradual water deficit based on visual evaluations. The growth substrate planted with the MDILs and the parental lines registered an average of 43–45% moisture content at day one of the experiment (Fig. 5a) which corresponds to the last day that the 4-week-old plants were watered. Moisture content in the potting mix declined sharply to less than 20% after a week of withholding water. Thereafter, moisture content gradually decreased and reached 0% after 4 weeks of non-watering. With the growth media at 5% moisture content, the leaves of the cultivated tomatoes LA3122, along with those of MDIL 3, 4I, 4II, 5, 7, 8I, 9 and 10 started wilting. At 0% moisture, the leaves of MDIL 8I, 11II and 12I started curling inwards while the rest of the plants, except for MDIL 2 and MDIL 11III, already started drying up. After 7 days at 0% moisture content, only MDILs 2 and 11III did not show any signs of wilting or inward curling of leaves, although a distinct delay in growth was observed. Additionally, the leaves of both MDIL 2 and MDIL 11III started turning into a darker shade of green when moisture content in the growth media decreased to 10% (Fig. 5b).

**Fig. 6:**
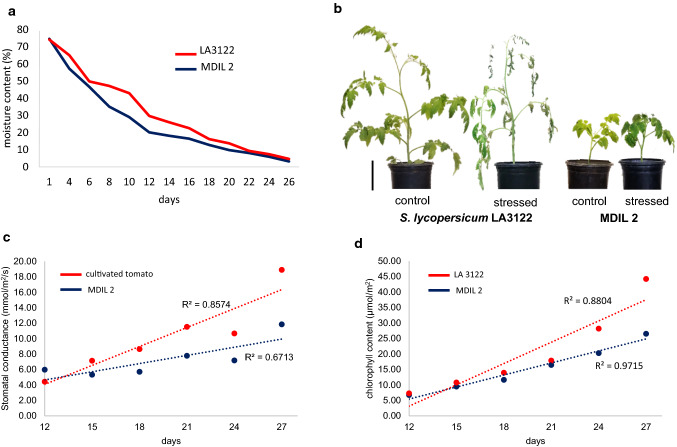
Morphological and physiological responses of MDIL 2 to drought stress. (**a**) Average moisture content of potting mix planted with tomato Acc. LA3122 and MDIL 2. Day 1 represent the last day the plants were watered to full saturation capacity. (**b**) Gross morphology of tomato Acc. LA3122 and MDILs 7 days after the moisture content of the potting mix reached 0%. (**c**) Average stomatal conductance and (**d**) chlorophyll content of tomato Acc. LA3122 and MDIL 2, respectively, measured when moisture content in the potting mix dropped to 25% after 12 days of withholding water. Bar = 10 cm

Based on the results of our preliminary screening for drought tolerance, replicated experiments to validate the tolerance of MDIL 2 to gradual water deficit were carried out. Moisture content in the growth media rapidly decreased to an average of 25% on day 12 of withholding water. Thereafter, moisture content decreased gradually until it reached 0% after 3.5 to 4 weeks of non-watering. Similar to our preliminary findings, the cultivated tomato started wilting when moisture content in the growth substrate reached approximately 5–6%. In contrast, MDIL 2 did not show any signs of wilting or curling of the leaves even after a week in potting mix with 0% moisture (Fig. 6a).

Under non-stressed conditions, stomatal conductance of MDIL 2 and LA3122 averaged 2.70 and 3.20 mmol/m^2^/s, respectively. These rates increased in both MDIL 2 and LA3122 with decreasing moisture content starting from day 12 of withholding water until the growth media registered 0% moisture content. Compared to the control plant, MDIL 2 exhibited a lower rate of increase, with only 67% of the variability in stomatal conductance explained by the decreasing moisture content in the potting mix. This is in contrast to the 86% of the variability in stomatal conductance in LA3122 that is explained by *r*^2^ model (Fig. 6b). Similarly, an increase in chlorophyll content was observed in both MDIL 2 and LA3122 as moisture content decreased from approximately 25% to 0%. The 97% variability in the chlorophyll content of MDIL 2 leaves was explained by the gradual loss in the moisture content of the potting mix, whereas 88% of the variability in the chlorophyll content of LA3122 is explained by the *r*^2^ model (Fig. 6c).

### Drought tolerance in MDIL 2 is putatively associated with a chromosome 4 introgression from *S. lycopersicoide*s

MDIL 2 has unique alien introgressions near the centromeric region of chromosome 1, distal end of the long arm of chromosome 4, and in the centromeric region of chromosome 7. Additionally, it also has a homozygous, non-parental introgression in chromosome 2. MDIL 11III, on the other hand, has a unique, non-parental introgression in the short arm of chromosome 5. All other introgressions detected in MDIL 2 and 11III are present in the rest of the MDILs.

A preliminary survey of gene annotations within the alien introgression in chromosome 4 of MDIL 2 identified approximately 1200 genes excluding those coding for hypothetical proteins and proteins with unknown functions. From these, we identified gene products and transcription factors that have been reported to be involved in plant responses to drought stress and that are related to the morphological (i.e., darkening of the green color of leaves) and physiological traits (i.e., stomatal conductance) observed in MDIL 2 and MDIL 11III in response to gradual water deficit. Examples of the structural and regulatory genes identified within the chromosome 4 introgression are the late embryogenesis abundant (LEA), stay-green, abscisic acid-insensitive 5 (ABI5) and ABSCISIC acid responsive elements-binding factor 1 (AREB-1) genes.

LEA proteins belong to a large group of hydrophilic and glycine-rich proteins in plants that are mainly expressed under drought conditions to protect cells (Magwanga et al. 2018). STAY-GREEN has been reported to maintain the green color of the leaves and sustain normal photosynthetic activity of sorghum, wheat, maize and barley under drought stress (Kamal et al. 2019). Both ABI5 and AREB1 are basic leucine zipper transcription factors that are involved in core ABA signaling under drought stress. In the presence of abscisic acid, AB15 regulates drought responses during seed development and early stages of seedling growth (Skubacz et al. 2016), while AREB1 expression has been found to be specific to vegetative tissues (Yanez et al. 2009; Orellana et al. 2010; Fujita et al. 2005). Under drought conditions, ABA signaling and transport are necessary requirements for stomatal closure to prevent moisture loss. Research is currently underway to determine the function of the identified ABA gene in the response of MDIL 2 to drought stress.

## Discussion

Elucidation of the genetic basis of observable phenotypes in exotic germplasm is a prerequisite to their effective utilization in target trait improvement in crops. MDILs are fertile lines having the full chromosome complement of the cultivated parent and limited chromosome segment introgressions from the wild parent. Because MDILs are mere ‘by-products’ of MAALs, their potential as genetic donors in introgressive breeding has been largely overlooked. In the current study, MDILs of *S. lycopersicoides* MAALs were genotypically and phenotypically characterized to provide basis for their applications in introgressive breeding programs. From a total of 38 MAAL progenies analyzed, 29 were identified as diploids although one line from MAAL 7 did not show any identifiable alien introgressions in its genome. The high percentage of diploid progenies identified segregating from the MAALs (76%) coincides with previous reports of 75–100% of *S. lycopersicoides* MAAL progenies having a chromosome composition of 2*n* (Chetelat et al. 1997).

Genotyping of the *S. lycopersicoides* MDILs demonstrated conserved patterns of alien introgressions among progenies segregating from the same MAAL as in the case of MDILs 2, 3, 4 and 8. Across MDILs, a degree of preferential transmission of specific chromosome segments was observed as exemplified by the only homozygous *S. lycopersicoides* segment in the long arm of chromosome 5 of MDILs 4, 8 and 11. In some cases, the systematic transfer of a chromosome segment occurs strictly within MDILs derived from the same MAAL. An example of this is the homozygous *S. lycopersicoides* introgression identified in the short arm of all MDILs that segregated from MAAL 5. Conversely, a distinct, non-transmission of alien introgressions was observed in chromosomes 3, 10 and 11 of all MDILs.

Preferential transmission of chromosomes or chromosome segments from a wild relative to a cultivated species is a phenomenon that has been reported in several crops such as cotton, wheat and rice. In cotton, four amplified fragment length polymorphism (AFLP) markers that are specific to *Gossypium sturtianum* were detected in all backcross progenies of the trispecies hybrids (*G. hirsutum* × *G. raimondii*) × *G. sturtianum* and (*G. raimondii* × *G. sturtianum*) × *G. hirsutum* used to improve gossypol content in seeds and whole cotton plants (Vroh Bi et al. 1999). Similarly, three SSR markers that are specific to *G. sturtianum* showed conserved transmission in all selected progenies of the trispecies hybrid (*G. hirsutum* × *G. raimondii*) × *G. sturtianum* (Benbouza et al. 2007). In rice, preferential transmission of *O. latifolia* chromosome segments in MDILs was marked by the conserved transmission patterns of a set of wild SSR, sequence-tagged sites, single nucleotide polymorphisms and indel loci on specific chromosomes (Angeles-Shim et al. 2014). In the case of both cotton and rice, the systematic transfer of marker loci is attributed to the higher pairing affinity in these regions between homeologous chromosomes of the wild and cultivated species used in the crosses. In a similar manner, the systemic replacement of tomato loci in the MDILs with chromosome segments from the wild nightshade species can also be explained by homeologous pairing between the divergent tomato and *S. lycopersicoides* genomes.

Aside from homozygous *S. lycopersicoides* introgressions, heterozygous and non-parental chromosome segments were also observed to be stably inherited in the MDILs. One possibility that could explain the persistence of heterozygous alleles in the MDILs is their ability to confer physiological and/or reproductive fitness such that plants acquiring the homozygous forms of either alleles will have lower fertility and hence lower chances of survival. On the other hand, the non-parental introgressions may be the result of genomic shock that was triggered by the initial hybridization between the two highly divergent genomes. Alternatively, the introgressions may have come from *S. pennellii* which was used to develop the bridging lines to generate the MAALs (Chetelat et al. 1997). Cloning and sequencing of the non-parental bands and comparing them with the target loci sequences from the different species used in the crosses will determine the nature of the off-type amplifications.

Phenotyping of the MDILs established a closer morphological resemblance of the introgression lines to the cultivated tomato than the wild parent. The rapid recovery of the recurrent phenotype in the MDILs after successive selfing of the MAALs is attributed to the extremely limited homeologous pairing between the chromosomes of the distantly related tomato and *S. lycopersicoides* (Stephens 1949; Rick 1971; Jena et al. 1994). Wide variability in qualitative traits including leaf and fruit characteristics was observed in the MDILs, with lines derived from the same MAAL but having different patterns of alien introgressions showing differences in the traits examined. Aside from the fruits of MDIL 4I resembling the fruits of MAAL 4, none of the MDILs had phenotypes that are exactly similar to those reported for the original MAALs (Chetelat et al. 1997), indicating the novelty of genetic variation captured by the MDILs. Similarly, the MDILs demonstrated variations in drought responses, with MDIL 2 and MDIL 11III showing tolerance to the stress. Physiological evaluation of MDIL 2 response to drought showed an increase in stomatal conductance and chlorophyll content in the leaves of the plant as moisture content in the growing media decreased.

Stomata are openings in the epidermis of leaves and stems that regulates photosynthesis and water movement through transpiration. Under water-limiting conditions, one of the first responses of the plant is to close its stomata to prevent moisture loss. Stomatal conductance measures the degree of the opening of the stomata and hence can be used as a physiological parameter to evaluate drought tolerance (Ahmad et al. 2014). Both LA3122 and MDIL 2 demonstrated an increase in stomatal conductance in response to drought, although MDIL 2 registered a lower rate of increase compared to the control tomato cultivar. The disparity in the increase in stomatal conductance between MDIL 2 and LA3122 may be indicative of a better water use efficiency in the former. In faba beans, drought-tolerant genotypes also recorded lower stomatal conductance compared to those that are drought-sensitive (Khan et al. 2007).

Aside from stomatal conductance, chlorophyll content is another physiological indicator of drought responses in plants. In barley and peanut, higher chlorophyll content in the leaves of plants under water-limiting conditions has been associated with tolerance to drought (Rong-hua et al. 2006; Arunyanark et al. 2008; Mohan et al. 2000). In the current study, an increase in chlorophyll content and change in color from yellow green to dark green were observed in MDIL 2 leaves in response to drought. LA3122 also registered an increase in chlorophyll content under drought conditions but unlike MDIL 2, the control plants were already wilted when moisture content in the growth media reached approximately 5%. These results indicate that under water-limiting conditions, an increase in chlorophyll content may not be as important if the plant is unable to prevent excessive water loss or use water more efficiently.

## Conclusion

*S. lycopersicoides* is a wild relative of tomato with known adaptation to a range of biotic and abiotic stresses. Pre-breeding programs targeting the utilization of this wild nightshade species as a donor in introgressive tomato breeding have led to the development of pre-breeding stocks that include early backcross lines, CSSLs and MAALs. While some of the early backcross lines and CSSLs have been used in breeding programs to improve disease resistance in tomato, the utilization of MAALs as donors in hybridization activities is extremely limited by the reproductive barriers associated with the genetic imbalance caused by the additional chromosomes.

Here, we report the molecular and morphological characterization of diploid introgression lines segregating from the progenies of *S. lycopersicoides* MAALs or MDILs. DNA marker profiling using SSRs and indels detected unique patterns of alien introgression in the genetic background of tomato and identified a degree of conserved chromosome transmission across the MDILs. The wide phenotypic variability that are distinctly different from the cultivated parent and even from those reported for the source MAALs indicates the acquisition of novel phenotypes by the MDILs through the alien chromosome introgressions as exemplified by drought tolerance in MDIL 2 and 11III. Such phenotype–genotype association was made possible by the uniformity of the genetic background of the MDILs which allowed the evaluation of the effects of the alien chromosome fragments on the morpho-agronomic traits of the plants. The strong correlation between phenotypes and introgressed chromosome fragments in these lines also eliminates the interference effects between QTLs, also known as ‘phenotypic noise’ that obscures the true phenotypic effects of individual loci. As a result, even QTLs with minor effects can also be easily detected. For better phenotype–genotype associations, the resolution of alien introgressions within the genome can be increased by sequencing each MDIL or significantly increasing the number of markers to genotype each line. Further screening of these exotic germplasm for resistance to pests and diseases, as well as to other abiotic stress, will facilitate the identification of novel genes from *S. lycopersicoides* that can be used for target trait improvement in tomato. Possibly the biggest advantage in exploiting the *S. lycopersicoides* MDILs in introgressive breeding is the elimination of the difficulties in generating crosses with tomato due to reproductive barriers.

## Supplementary Information

Below is the link to the electronic supplementary material.

Supplementary file 1 (XLSX 32 kb)

## References

[CR1] Ahmad M, Zaffar G, Razvi S, Dar Z, Habib B (2014). Resilience of cereal crops to abiotic stress: a review. Afr J Biotech.

[CR2] Angeles-Shim RB, Shim J, Vinarao RB, Lapis RS, Singleton J (2020). A novel locus from the wild allotetraploid rice, Oryza latiolia desv confers bacterial blight (Xanthomonas oryzae pv oryzae) resistance in rice (O sativa L). PLoS ONE.

[CR3] Angeles-Shim RB, Vinarao RB, Marathi B, Jena KK (2014). Molecular analysis of Oryza latifolia Desv (CCDD genome)-derived introgression lines and identification of value added traits for rice (O sativa L) improvement. Heredity.

[CR4] Arunyanark A, Jogloy S, Akkasaeng C, Vorasoot N, Kesmala T, Nageswara Rao RC, Wright GC, Patanothai A (2008). Chlorophyll stability is an indicator of drought tolerance in peanut. J Agro Crop Sci.

[CR5] Bai Y, Mattoo A, Handa A (2017). Developments in tomato breeding: conventional and biotechnology tools. Achieving sustainable cultivation of tomatoes.

[CR6] Bai Y, Lindhout P (2007). Domestication and breeding of tomatoes: what have we gained and what can we gain in the future?. Ann Bot.

[CR7] Benbouza H, Diouf FBH, Baudoin JP, Mergeai G (2007) Preferential transmission of *Gossypium sturtianum* chromosome fragments in the progeny of [(*G. hirsutum x G. raimondii*)^2^ x *G. sturtianum*] trispecific hybrid. In: Proceedings of the World Cotton Research Conference-4. Lubbock, Texas,USA. 10–14 September 2007

[CR8] Besho-Uehara K, Furuta T, Masuda K, Yamada S, Angeles-Shim RB, Ashikari M, Takashi T (2017). Construction of rice chromosome segment substitution lines harboring *Oryza barthii* genome and evaluation of yield-related traits. Breed Sci.

[CR9] Canady MA, Meglic V, Chetelat RT (2005). A library of *Solanum lycopersicoides* introgression lines in cultivated tomato. Genome.

[CR10] Chetelat RT, Cisneros P, Stamova L, Rick CM (1997). A male-fertile *Lycopersicon esculentum* x *Solanum lycopersicoides* hybrid enables direct backcrossing to tomato at the diploid level. Euphytica.

[CR11] Chetelat RT, Rick CM, Cisneros P, Alpert KB, DeVerna JW (1998). Identification, transmission and cytological behavior or *Solanum lycopersicoides* Dun. monosomic alien additional lines in tomato (*Lycopersicon esculentum* Mill). Genome.

[CR12] Davis J, Yu D, Evans W, Gokirmak T, Chetelat RT, Stotz H (2009). Mapping of loci from *Solanum lycopersicoides* conferring resistance or susceptibility to *Botrytis cinerea* in tomato. Theor Appl Genet.

[CR13] FAOSTAT (2020) Production/Yield quantities of Tomatoes in World + (Total). Food and Agriculture Organization. http://www.fao.org/faostat/en/#data/QC/visualize. Accessed 7 July 2020

[CR14] Fujita Y, Fujita M, Satoh R, Maruyama K, Parvez MM, Seki M, Hiratsu K, Ohme-Takagi M, Shinozaki K, Yamaguchi-Shinozaki K (2005). AREB1 is a transcription activator of novel ABRE-dependent ABA signaling that enhances drought stress tolerance in *Arabidopsis*. Plant Cell.

[CR15] Furuta T, Uehara K, Angeles-Shim RB, Shim J, Nagai K, Ashikari M, Takashi T (2016). Development of chromosome segment substitution lines (CSSLs) harboring O nivara segments and evaluation of yield-related traits. Breed Sci.

[CR16] Gradziel TM, Robinson RW (1989). *Solanum lycopersicoides* gene introgression to tomato, *Lycopersicon esculentum*, through the systematic avoidance and suppression of breeding barriers. Sex Plant Reprod.

[CR17] Gur A, Zamir D (2004). Unused natural variation can lift yield barriers in plant breeding. PLoS Biol.

[CR18] http://marker.kazusa.or.jp/Tomato/ Accessed 20 March 2019

[CR19] IPGRI (International Plant Genetic Resources Institute) (1996) Descriptors for Tomato (*Lycopersicon* spp.). IPGRI, Rome, Italy

[CR20] Jena KK, Khush GS, Kochert G (1994). Comparative RFLP mapping of a wild rice, *Oryza officinalis*, and cultivated rice *O. sativa*. Genome.

[CR21] Kamal NM, Gorafi YSA, Abdelrahman M, Abdellatef E, Tsujimoto H (2019). Stay-Green trait: a prospective approach for yield potential, and drought and heat stress adaptation in globally important cereals. Int J Mol Sci.

[CR22] Khan H, Link R, Hocking W, Stoddard T (2007). Evaluation of physiological traits for improving drought tolerance in faba bean (Vicia faba L). Plant Soil.

[CR23] Khush GS (2010). Trisomics and alien addition lines in rice. Breed Sci.

[CR24] Li J, Liu L, Bai Y, Zhang P, Finkers R, Du Y, Visser RGF, Van Heusden AW (2011). Seedling salt tolerance in tomato. Euphytica.

[CR25] Li R, Guo P, Michael B, Stefania G, Salvatore C (2006). Evaluation of chlorophyll content and fluorescence parameters as indicators of drought tolerance in barley. Agric Sci China.

[CR26] Magwanga RO, Lu P, Kirungu JN, Lu H, Wang X, Cai X, Zhou Z, Zhang Z, Salih H, Wang K, Fang L (2018). Characterization of the late embryogenesis abundant (LEA) proteins family and their role in drought stress tolerance in upland cotton. BMC Genet.

[CR27] Mangat PK, Gannaban RB, Singleton JJ, Angeles-Shim RB (2020). Development of PCR-based, genetic marker resource for the tomato-like nightshade relative, *Solanum lycopersicoides* using whole genome sequence analysis. PLoS ONE.

[CR28] Mohan MM, Laxmi NS, Ibrahim SM (2000). Chlorophyll stability index (CSI): its impact on salt tolerance in rice. Int Rice Res Notes.

[CR29] Miller JC, Tanksley SD (1990). RFLP analysis of phylogenetic relationships and genetic variation in the genus *Lycopersicon*. Theor Appl Genet.

[CR30] Murray MG, Thompson WF (1980). Rapid isolation of high molecular weight plant DNA. Nucleic Acids Res.

[CR31] Narain A, Kar MK, Kaliaperumal V, Sen P (2016). Development of monosomic alien addition lines from the wild rice (*Oryza**brachyantha* A. Chev. et Roehr.) for introgression of yellow stem borer (*Scirpophaga incertulas* Walker.) resistance into cultivated rice (*Oryza**sativa* L.). Euphytica.

[CR32] Orellana S, Yañez M, Espinoza A, Verdugo I, Gonzáles E, Ruiz-Lara S, Casaretto J (2010). The transcription factor SlAREB1 confers drought, salt stress tolerance and regulates biotic and abiotic stress-related genes in tomato. Plant Cell Environ.

[CR33] Perez de Castro A, Diez MJ, Nuez F (2011) Evaluation of a subset of *Solanum lycopersicoides* introgression lines for resistance to *Tomato yellow leaf curl* disease. In: Proceedings of the XVII Eucarpia Meeting Group-Tomato, Malaga Spain, 17

[CR34] Phills BR, Provvidenti R, Robinson RW (1977). Reaction of *Solanum lycopersicoides* to viral disease of tomato. Rep Tomato Genet Coop.

[CR35] Phills BR, Robinson RW, Shail JW (1977). Reaction of *Solanum lycopersicoides* to fungal diseases and nematodes. Rep Tomato Genet Coop.

[CR36] Rick CM, Chetelat R (1995) Utilization of related wild species for tomato improvement, First International Symposium on *Solanacea* for Fresh Market. Acta Hortic 412:21–38. 10.17660/ActaHortic.1995.412.1

[CR37] Rick CM (1971). Further studies on segregation and recombination in backcross derivatives of a tomato species hybrid. Biol Zentrabl.

[CR38] Rick CM (1976) Tomato In: Simmonds NW (eds) Evolution of crop plants. Longman, London

[CR39] Shim J, Torollo G, Angeles-Shim RB, Cabunagan RC, Choi IR, Yeo US, Ha WG (2015). *Rice tungro spherical virus* resistance into photoperiod-insensitive japonica rice by marker-assisted selection. Breed Sci.

[CR40] Shim RA, Ashikari M, Angeles ER, Takashi T (2010). Development and evaluation of *Oryza glaberrima* Steud chromosome segment substitution lines in the background of *O. sativa* subsp. *japonica* cv. Koshihikari Breed Sci.

[CR41] Skubacz A, Daszkowska-Golec A, Szarejko I (2016). The role and regulation of ABI5 (ABA-Insensitive 5) in plant development, abiotic stress responses and phytohormone crosstalk. Front Plant Sci.

[CR42] Stephens SG (1949). The cytogenetics of speciation in *Gossypium*; selective elimination of the donor parent genotype in interspecific backcrosses. Genetics.

[CR43] Vroh Bi I, Maquet A, Baudoin JP, Du Jardin P, Jacquemin JM, Mergeai G (1999). Breeding for “low-gossypol seed and high-gossypol plants” in upland cotton: analysis of tri-species hybrids and backcross progenies using AFLPs and mapped RFLPs. Theor Appl Genet.

[CR44] Wang X, Wang Y, Wang C, Yu C, Yu C, Feng S, Zhao T, Zhao B (2016). Characterization of eleven monosomic alien addition lines added from *Gossypium anomalum* to *Gossypium hirsutum* using improved GISH and SSR markers. BMC Plant Biol.

[CR45] Wolfe S, Yakir D, Stevens MA, Rudich J (1986). Cold temperature tolerance of wild tomato species. J Amer Soc Hort Sci.

[CR46] Yáñez M, Cáceres S, Orellana S, Bastias A, Verdugo I, Ruiz-Lara S, Casaretto JA (2009). An abiotic stress-responsive bZIP transcription factor from wild and cultivated tomatoes regulates stress-related genes. Plant Cell Rep.

[CR47] Zhang L, Li Z, Li J, Wang A (2013). Ectopic overexpression of *Sscbf1*, a CRT/DRE-binding factor from the nightshade plant *Solanum lycopersicoides*, confers freezing and salt tolerance in transgenic Arabidopsis. PLoS ONE.

[CR48] Zong YY, Liu L, Li T, Sayed-Rashid AS, Zhou LX, Sun YY, Zheng Z, Zheng QG, Fan SY, Li JM (2012). Mapping of QTLs conferring the resistance to Tomato yellow leaf curl virus (TYLCV) in *Solanum lycopersicoides*. Acta Hortic Sin.

